# Study protocol: evaluation of specialized outpatient palliative care in the German state of Hesse (ELSAH study) – work package I: assessing the quality of care

**DOI:** 10.1186/s12904-018-0363-8

**Published:** 2018-10-02

**Authors:** Katrin Kuss, Hannah Seipp, Dorothée Becker, Stefan Bösner, Antje Erler, Dania Gruber, Michaela Hach, Lisa R Ulrich, Jörg Haasenritter

**Affiliations:** 1 Department of General Practice/ Family Medicine, Philipps-University Marburg, Karl-von-Frisch-Strasse 4, 35032 Marburg, Germany; 2 Professional Association of Specialized Outpatient Palliative Care in Hesse, Wiesbaden, Germany; 3 Institute of General Practice, Goethe-University Frankfurt, Frankfurt, Germany

**Keywords:** Palliative care [MeSH], Quality of health care [MeSH], Outcome assessment [MeSH], Validation, Implementation, Evaluation, Mixed methods, Study protocol

## Abstract

**Background:**

In Germany, patients suffering from life-limiting conditions are eligible for specialized outpatient palliative care (SOPC). Evaluation of the quality of this service lacks currently integration of patient-relevant outcomes. There is also no scientific consensus how to prove quality of care in the special context of SOPC adequately. Existing quality reports are primarily based on descriptive structural data which do not allow for estimation of process quality or result quality. The ELSAH study (‘Evaluation of Specialized Outpatient Palliative Care in the German state of Hesse’) aims to choose - or, if necessary, to adopt - to evaluate and to implement a suit of measures to assess, evaluate and monitor the quality of specialized, home-based palliative care.

**Methods:**

All 22 SOPC teams providing their services in the state of Hesse, Germany, participate in the ELSAH study. The study is divided in two phases: a preparation phase and a main study phase. Based on the findings of the preparation phase we have chosen a preliminary set of instruments including the Integrated Palliative Outcome Scale, Views on Care, Zarit Burden Interview, Phase of Illness, Goal Attainment Scaling, Eastern Cooperative Oncology Group Performance Status, Consumer Quality Indices Palliative Care and Sense of Security in Care. During the main study phase, we will use a mixed-methods approach to evaluate the instruments’ psychometric properties (reliability, validity, feasibility and practicability), to identify barriers, facilitators and limitations of their routine use and to explore how their use affects the care within the SOPC setting.

**Discussion:**

At the end of this study, an outcome- and patient-centered, validated measurement approach should be provided, adapted for standardized evaluations in SOPC across patient groups, palliative care services and regions nationwide. The standardized application of instruments should allow for making valid statements and comparisons of health care quality in SOPC based on process- and outcome-evaluation rather than relying on structural data only. Moreover, the instruments might directly influence the care of patients in palliative situations.

**Trial registration:**

German Clinical Trials Register (DRKS-ID: DRKS00012421).

## Background

In Germany, people with non-curable, progressive, and life-limiting diseases, who show complex symptoms, burdens and care requirements, are entitled to receive specialized outpatient palliative care (SOPC) [1]. Multi-professional teams (physicians, palliative care nurses, and optionally psychologists, social workers etc.) provide home-based care, including medical and nursing care, a 24/7 on-call service, psychosocial support and coordination of care in cooperation with primary care givers. SOPC can be provided as counseling of patient and care givers, coordination of care or additional supportive or full care. The aim of SOPC is to foster the patient’s autonomy, quality of life and to allow a decent living and dying in his/her familiar home environment. In 2015, in the German state Hesse 66,532 persons died and 6495 persons received a first time prescription of SOPC [1]. The basis of SOPC provision is regulated by a directive of the Federal Joint Committee (G-BA), the highest decision-making body of the joint self-government of physicians, dentists, hospitals and health insurance funds in Germany [2]. Regular reports of the G-BA on the evaluation of the SOPC directive mainly comprise structural data, such as number of prescriptions, costs, number of care provider teams and their skills mix [3]. While a more comprehensive and more in-depth insight in the quality of care might be strongly desirable, it remains a challenge and actually there is no scientific consensus how to evaluate or monitor quality of care in the special context of SOPC adequately on the basis of standardized data [4]. It seems apparent that there are divergent views which should be considered (primarily patients and persons close to them, but also professionals of palliative care services, to some extent health care system e.g. health insurances or policymakers). In this context, outcome measurement and especially patient-reported outcome measurement (PROM) has increasingly gained attention to assess the quality of palliative care [5].

Outcome measurement in palliative care has to fulfil several requirements [6, 7]: Since palliative care is not restricted to patients with oncologic diagnoses, measurement should be universally applicable across different populations. It should be suited to the aims and important domains of the care being delivered. It should address the needs of patients as well as the needs of unpaid caregivers. Whilst the perspective of those affected plays an important role in all areas of health care, this specifically applies to palliative care. Patient-reported or at least patient-centered outcome measures are strongly recommended to be applied, if possible, in end-of-life care [8]. Nevertheless, especially regarding the situation of patients with progressive diseases and psychic strain, measures must proof to be practical and efficient. Moreover, patient-centered outcome measures rated by proxies – persons close to the patient or professionals – should be taken into consideration as an alternative to patient-reported measures. The measures should have sound psychometric properties, namely reliability, validity, and sensitivity to change.

Outcome measurement fulfils different tasks [6]: it allows to evaluate and to manage the care of an individual patient and supports clinical decision making. Additionally, aggregate data gives professionals the possibility for self-reflection, aiming for improvement of the quality of care. Typically, a set of measures is necessary to evaluate quality of care.

Against this background and taking the situation of the German state of Hesse as an example, the work package I of the ELSAH study (‘Evaluation of Specialized Outpatient Palliative Care in the German state of Hesse’) aims to choose - or, if necessary, to adopt - to evaluate and to implement a suit of measures to assess, evaluate and monitor the quality of specialized, home-based palliative care. This paper presents the study protocol of work package I.

The ELSAH-study comprises a second work-package. In this work package, the special needs of pediatric patients (children, adolescents, and young adults) and their families, and how SOPC for children differs from SOPC for adults, will be examined. The Institute of General Practice of Goethe-University Frankfurt, Germany, is responsible for the second work package. The study protocol for work package II has been published separately [9].

## Methods and design

The ELSAH-study takes place in the state of Hesse, Germany. There are 22 SOPC teams, who provide their services to the approximately 6 million inhabitants. The teams are organized within the Professional Association of SOPC of Hesse (Fachverband SAPV Hessen e.V. [10]), which has the lead of the ELSAH research cooperation. Within this association, each team agreed on sharing a common quality management system and on developing a common documentation system.

Each team provides standardized and non-standardized minimum data on each case on an annual basis with 6.500 cases in 2015. These structures facilitate the recruitment of study participants (professionals and those affected) and the planned statewide implementation and evaluation of the quality instrument set.

The study started in April 2017 and will end in March 2020; the recruitment of participants for the entire project started in May 2017 and will end in December 2019.

**Fig. 1:**
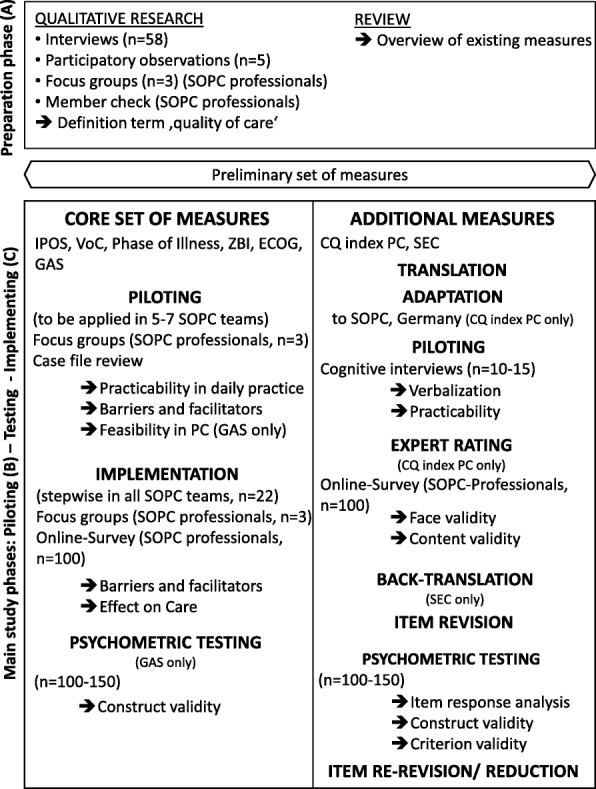
Flow of study

The study is divided in two phases: a preparation phase and a main study phase. The preparation phase has been finished and was the prerequisite for choosing a set of preliminary instruments. Aims of the main study are to pilot, adopt, test and implement the instruments within the SOPC setting. Figure 1 shows the study work flow.

### Preparation phase

The aim of the preparation phase was to further define the term “quality of care” in the context of SOPC. Therefore, the research focus to explore the concept of successful care in the context of SOPC took into account the physical, psychological, social, and spiritual dimension. Moreover, a preliminary set of instruments was chosen and adapted. Preliminary results of the first study phase are depicted in the following paragraphs to facilitate the comprehensibility of the protocol regarding the main study phase.

Guided semi-structured interviews were conducted with health care professionals (experts working in a SOPC-team, hospice services, chaplains, general practitioners; *n* = 30) as well as with adult patients officially included in SOPC, persons close to them, and bereaved persons (*n* = 28). Potential participants were recruited by SOPC-teams, who contacted potential interview partners. In case of informed consent, the study team arranged an appointment at the participant’s current residence for the interview. There were several possibilities for the conduction of interviews: a patient only, the patient and a person close to her/him together or one after another, or only a person close to the patient (or a bereaved one). The choice depended on family/social relations, the physiologic and psychologic ability and willingness, and particularly on the patient’s intention. The sample size of this qualitative study phase was based on the principles of ‘Theoretical Sampling’: this means to reach broad and contrasting opinions until further concepts were scarcely to be expected to emerge from the interviews. To enrich the information gathered by single person interviews, three focus groups with care providers from SOPC-teams were conducted. To get deeper insights into home-based palliative care, research team members accompanied different SOPC-teams on a typical working day (participant observation). All kinds of regions (urban, suburban, rural) of Hesse were within the scope. The researchers took field notes of their impressions in terms of a ‘small ethnography’.

Interviews and focus groups were conducted by three researchers (KK, HS, SB) experienced in handling communication with patients and relatives in critical situations. Nevertheless, they attended a training by a licensed course instructor for palliative care (MH) to anticipate and handle potential critical interview situations. Each interview was transcribed verbatim following established standards [11]. The interview transcripts and the written field notes of the participant observations were analyzed using content-analytic methods [12], software-assisted by MAXQDA version 18 [13].

The focus group discussions were videotaped. For the analysis, a thematic precoding of the videos was done assisted by MAXQDA. Additionally, the videos were analyzed in a research-team process which was thematically based on the Focusgroup Illustration Map method [14] and on triangulation with the interview and observation categories. Data analysis was done parallel to the interviews following the principle of ‚Grounded Theory‘ [15]. All results derived of qualitative data built a sequential Mixed Methods Designs [16], and allowed the triangulation of results derived by different qualitative methods [17].

Preliminary results of the preparation phase indicated that there are three central constructs: symptom control under SOPC, sense of security, and comprehensive care. The final results that will derive from qualitative data analysis will be presented in a separate paper in accordance with accepted criteria for presenting qualitative data. Results also will be presented to SOPC teams within statewide periodic quality circles.

Additionally, a comprehensive overview of existing instruments for measuring outcome quality was compiled. Special focus was set on patient-reported outcome measures with approved psychometric properties (reliability, validity, sensitivity of change, practicability), which seem to be applicable or adaptable in outpatient palliative care in the German health care system.

As the main result of the preparation phase, a preliminary set of instruments to evaluate and monitor the ‘quality of care’ in SOPC was determined. The draft was presented to a national expert committee, including tenure professors and care professionals for palliative medicine, patients’ mandatories, medical ethicists, and others. At the expert-workshop that took 2 days, the instrument-sets were discussed and accepted. Not only are the tools expected to document quality of care, but also to assess actual needs for care from patient’s perspective and facilitate its prioritization. By a continuous assessment and respective reaction to changed needs and priorities, patients’ care should be improved.

### Main study: piloting, testing and implementing the instruments

Two sets of instruments emerged from the results of the preparation phase – a core-set of measures (Table 1) which will be applied in daily routine in order to assess and monitor relevant outcomes of care. An additional set of measures (Table 2) will be applied recurrently to evaluate the quality of the structure and processes of care from user’s perspective (patients and persons close to them). It seems suitable to separate the description of methods into the two outcome sets.

**Table 1:** Core set of measures

Measure	Description	Application time
Phase of Illness	The measure describes the stage in the patient’s illness and the suitability of the care plan according to the needs of the patients and their unpaid care givers. The scale classifies the patient into one of 5 possible stages: stable, unstable, deteriorating, dying and deceased [34, 35]. The instrument is recommended within the Outcome Assessment and Complexity Collaborative (OACC) suite of measure [18].	On admission and on subsequent assessments if changes occur.
Eastern Cooperative Oncology Group Performance Status (ECOG)	The 6-grade expert-rated ECOG describes the patient’s level of physical functioning/disability in terms of daily activities [36].	On admission and on subsequent assessments if changes occur.
Integrated Palliative care Outcome Scale (IPOS)	IPOS is based on the Palliative care Outcome Scale (POS) which has been validated within different PC settings [37] and is one of the most frequently used outcome measures in palliative care [38]. It captures the patient’s most important concerns (symptoms, information needs, practical concerns, anxiety of patients’ and care givers’ and overall feeling of being at peace). The different domains are scored using a 5-grade likert scale. The self-report of the patient is preferred, however, proxy versions for professionals and unpaid care givers are available. The instrument is recommended within the OACC suite of measure [18].	On admission and subsequently after about 5–10 days AND ≥ 3 appointments, at least once in the further course and if changes occur.
IPOS Views on Care (VoC)	IPOS Views on Care is based on the St. Christopher’s Index of Patient Priorities (SKIPP) tool [39] and can be used as a supplement to IPOS [40]. The measure captures the patients’ view on the impact the palliative care service has on their quality of life and their principal problems. Patients rate their overall quality of life for the time before admission to the palliative care service and the time after admission. It includes four items. The instrument is recommended within the OACC suite of measure [18].	On admission and subsequently after about 5–10 days AND ≥ 3 appointments, at least once in the further course and if changes occur.
Zarit Burden Interview (ZBI)	The shortened 6-item interview form recommended for palliative care [41] is opted. The questions inquire the amount in which the unpaid caregiver is negatively affected by his/her role on a 5-point Likert-type scale. For this study an additional global item is added to serve as anchor question for creating a classification able to interpret the total score. The instrument is recommended within the OACC suite of measure [18].	On admission and subsequently at least once in the further course.
Goal Attainment Scaling (GAS)	GAS reflects a process to create and to reassess individual scales for measuring the grade of care success goal-oriented [19]. This process should be applied in addition to standardized outcome measures and has potential to reveal and to prioritize patient-relevant needs or wishes. For each identified goal indicators on a 5-point matrix in grades from − 2 to + 2 are framed and continuously reassessed over the course of care. So far, experiences in the palliative care field are lacking.	Each appointment.

**Table 2:** Additional set of measures

Measure	Description	Application time
Consumer Quality Index Palliative Care (CQ-index PC) Patients	The CQ-index PC is a standardized questionnaire developed and validated by the Dutch health ministry, that aims to measure the quality of palliative care from the view of those affected; there is a patient version [21] and a version for bereaved ones applied some weeks after bereavement [22]. Unlike to other scales the object of rating is not satisfaction; with regard to several aspects of care each item is rated for one thing how the aspect was experienced, for another thing how important this aspect is to that person. The CQ-index PC can be used to assess the quality of palliative care in various settings. The original version of the CQ-index PC for Patients includes 88 items (32 experience items, 32 importance items and 24 on background characteristics). Items are clustered in several domains (e.g. care for physical well-being, care for psychological wellbeing, care for spiritual well-being, respecting independence, respecting privacy, information, expertise of caregivers). The original version of the CQ-index PC for bereaved ones includes 102 items (34 experience items, 34 importance items and 34 on background characteristics.	Once in the second week of care.
Consumer Quality Index Palliative Care (CQ-index PC) Bereaved		Once 6–24 weeks post mortem.
Sense of Security Patients (SEC-P)	Sense of security in care seems to be a prerequisite to enable home-care for patients with advanced disease and their unpaid caregivers [42]. The SEC is so far the only known instrument measuring the patients’ and relatives’ sense of security in palliative care. The two versions of the questionnaire, one for patients (15 items) [23], one for relatives (17 items) [24], are available in a validated Swedish version. Each version consists of three sub scales.	Once after about 5–10 days AND ≥ 3 appointments.
Sense of Security Relatives (SEC-R)		Once after about 5–10 days AND ≥ 3 appointments.

#### Core set of measures – piloting and implementing

Most of the instruments considered here – like the Integrated Palliative Outcome Scale (IPOS), Phase of Illness and the Zarit Burden Interview (ZBI) – have been validated within diverse palliative care settings and have been recommended for regular use in palliative care [18]. Regarding these instruments we aim to evaluate the feasibility and practicability, to identify barriers, facilitators, and limitations of their routine use and to explore how their use affects the care within the SOPC setting.

Besides these instruments which are established in palliative care, we will use Goal Attainment Scaling (GAS) [19], which originates in the mental health field, for use in palliative care. GAS is a procedure to formulate individualized goals and objectively evaluate them. Contrary to standardized pre-formulated assessment tools each GAS chart is individually created by an interview process of a health professional and the patient [20]. Alternatively, persons close can assist or charts can be created within the team. The individualization is not only referring to the goal, but also to the indicator chosen whereby a five-point scale ranging from − 2 to + 2 is built to objectively rate whether the actually attained level refers to the expected level of achievement (score ‘0’) or to a much or somewhat better (score ‘+ 2’ or ‘+ 1’) or much or somewhat worse (score ‘-2’ or ‘-1’) level. GAS is meanwhile established in psychology, physical therapy, and occupational rehabilitation, for example in neurology, geriatric medicine, or chronic pain rehabilitation. Using GAS could enrich palliative care as a valuable supplementary tool to meet the requirements of individualization. Although goals are already addressed, GAS advances the process of goal setting, prioritization, pooling of resources, and evaluation more systematically. Moreover, the joint process between the health professional and the patient facilitates communication on realistic perceptions and can help to identify further needs. This also pertains to the meetings within the team. By the transformation of the scores of individually set goals into a single, aggregated t-score [19], GAS visualizes the beneficial effect of SOPC and allows for objective comparison across patients with very varying conditions. To validate GAS for use in palliative care, practicability, content-validity, construct-validity, reliability, and sensitivity to change will be evaluated by individual case assessments in combination with Consumer Quality-indices PC (CQ-indices PC) and Sense of Security (SEC), see also below. To our knowledge, there have been no studies evaluating and reporting the use of GAS within a palliative care setting. For the piloting of the instruments starting in May 2018, five to seven SOPC teams will be chosen sequentially on a voluntarily basis, taking into account criteria to reach a broad diversity, regarding regional differences and the organizational structure of the teams. Participating teams, consisting of 6–20 professionals each, will be trained in study procedures and practical application of instruments in daily routine. The teams will be supervised for a period of 2-months of participation.

After the piloting phase, adaptations of instruments will be considered. In the final study phase, we will implement the instruments successively in all SOPC-teams (*n* = 22) in the state of Hesse.

To evaluate the feasibility, practicability, and utility we will use a mixed-methods approach; we will analyze data gathered with these instruments in combination with data gathered within routine documentation such as gender, age, disease, symptoms, therapeutic management, structural details of the care situation, and at the close of the care process an evaluation of problem solving achieved, level of satisfaction rated by professionals, and the place of death. In addition, we will conduct focus group discussions (2–3 groups with 6–8 health care professionals each), supplemented by continuously given feedback of SOPC professionals in structured and unstructured manner during the pilot-phase, and feedback gathered by a semi-structured online-survey of SOPC team members (*n* = 60–100).

#### Additional set of measures: adaptation, validation and evaluation

The additional set of measures includes the Consumer Quality Indices Palliative Care (CQ-indices PC) [21, 22] and the Sense of Security in Care (SEC) [23, 24]. The methodological approach is basically the same for both of the instruments but differs slightly in the following way: regarding the SEC we aim to validate a German version. With respect to the CQ-indices PC our primary aim is not to validate the original questionnaires. The CQ-indices PC were developed to assess a broad range of health services. However, we will focus on a very specific setting (SOPC). Moreover, some items of CQ-indices PC are irrelevant in the German setting, e.g. in the original version patients are asked if health professionals provide information on euthanasia which is strictly forbidden in Germany. Therefore, CQ-indices PC will be used as a pool of items to derive a tool for the specific setting of SOPC.

German health professionals fluent in Dutch or Swedish language will translate all questionnaires into German. Study team members will adapt the wording of the questionnaires according to cross-cultural and setting-specific factors.

Adult SOPC patients, their unpaid caregivers/relatives and bereaved ones (*n* = 5–10 each) are encouraged for a cognitive-pretesting of the questionnaires [25, 26], including ‘think aloud’ and probing techniques to assess practicability and to identify unclear verbalization [27, 28].

To assess content validity of the items of the CQ-indies PC, professionals of all SOPC teams in Hesse and national palliative care experts will be invited to rate the appropriateness of all items to assess the quality of care within the setting of SOPC. For this purpose, a four-grade likert-type scale will be used.

Based on the results, item reduction (CQ-indices PC) and re-wording (CQ-indices PC and SEC) will be considered. Removal of an item will be considered if the median of the professionals’ rating is below the three. Complementary, participants will be asked to suggest relevant additional items. The resulting version of SEC will be translated back into the original language and potential differences will be discussed with the authors. For the CQ-indices PC, backward translation is not mandatory due to the fact that we do not aim for an one-to-one adaptation of the original instrument.

To test psychometric properties of both instruments, we will conduct a field test among *n* = 100–150 cases. Study participants (patients or their unpaid caregivers/relatives or their bereaved ones) have to be at least 18 years old, provided care by a SOPC team, fluent in German to complete the questionnaire and willing to participate in our study. Descriptive analyses (skewness of distributions, floor−/ceiling-effects, missing values, high correlation in item-pairs, low relevance for patients and persons close to them) will indicate problematic items.

To assess internal consistency, we calculate Cronbach’s alpha among scales and sub-scales. For all cases, we will combine data gathered with both measure sets tested within this study with additional data routinely documented for each patient included in SOPC in Hesse. In order to assess construct validity, we will evaluate pre-specified assumptions of correlations of the different constructs within our data [29]. For example: we assume that a change in the symptom burden as measured by IPOS will correlate with the patient’s assessment of the quality of care regarding his or her physical wellbeing as measured by the CQ-index PC. Due to the fact, that we will test a series of assumptions within a relatively small sample size, we will not perform formal statistical hypothesis testing, which would be prone to multiple testing. Instead of *p*-values we will provide correlations coefficients and the respective 95% confidence intervals. For SEC, the development studies showed three-component structures of both, the patient and the relatives version [23, 24]. We will use principal component analysis to confirm these structures.

As there is no accepted instrument as reference standard for measuring criterion validity, individual case assessments by an expert panel will serve as reference standard. All obtainable data of a patient case will be included. The overall quality of care will be rated by an independent expert panel, consisting of at least one member of the research team and one palliative care-expert. A six-point Likert-type scale ranging from 1 (very good quality) to 6 (unsatisfactory quality) will be applied; this rating will serve as reference standard for the CQ-indices PC.

### Ethical considerations

This study is based on the participation of a vulnerable group of patients and their relatives. To minimize strain, the study team made several provisions. Patients and persons close to them will be elected and invited by cooperating palliative care teams. Interview appointments will also be coordinated in close cooperation with the responsible palliative caregiver. Shortly before the interview, the patient and the allocating caregiver will again be called to ascertain whether the patient is able to do the interview. Patients can determine overall conditions, e.g. which person should be present while the interview. All participants have the possibility to cancel, pause or quit the interview as well as the study participation at all times. If necessary, members of the responsible palliative care team can be called in for support. Moreover, every interviewer is experienced in dealing with patients and relatives in exceptional situations. Nevertheless, the interviewers will be trained to detect and adequately react to problematic situations. Furthermore, there is the opportunity for clinical supervision in case of psychological burden.

All participants have to give signed informed consent before participation. All data will be pseudonymized or, if possible, anonymized for data analysis and for publication. The master database of all interview data, including cognitive interviews, is stored by the principal investigator of the Department of General Practice in Marburg. The master databases for individual case assessments will be kept by each cooperating palliative care team, data will be transferred pseudonymized to the study team. All master databases will be deleted at the end of the study, all other study documents will be deleted after 10 years. Individual Online surveys will be anonymous.

The study was approved by the local ethics committee of the Marburg university clinic (ref. 34/17, preparation phase; and ref. 47/18, main study phase) and is in accordance with the Declaration of Helsinki 1975, revised Fortaleza 2013. If relevant modifications of the study protocol occur, we will announce these to the funder, to the ethics committee by an amendment, and within the trial register.

## Discussion

The aim of this multistage project is to develop, evaluate, and implement a measurement approach for the evaluation of the quality of care (process and outcomes) and for guiding the care process based on individual needs and preferences in specialized outpatient palliative care in Hesse, Germany.

Actually, there is a broad array of instruments, which capture different aspects of care from the perspective of those affected [30]. The requirements for the use of each instrument are challenging. Moreover, resources, e.g. physical or psychological, are limited in health care in general and particularly in palliative care. On the one hand, instruments have to generate a comprehensive view – covering all relevant aspects inherent to SOPC. On the other hand, the capabilities of persons in end-of-life situations are limited. Therefore, aiming to apply PROMs as a basis, the approach of assessing process- and outcome-quality in SOPC must be reasonable and feasible [7]. The sample of patients in SOPC varies in diagnoses and the needs of the individual patient. Not withstanding, evaluation should allow comparison of outcomes across divergent conditions despite concurrent individuality. The needs of a patient are likely to change over a limited course of care. Therefore, an instrument must be sensitive to changes and furthermore allow adjustment of individual prioritization. The successful implementation of such instruments in daily practice is affected by several determinants that have to be taken into consideration [31]. In the implementation phase of the ELSAH study, every team in Hesse will be included to discover problems, refine strategies, and enhance implementation strategies.

Empathy and awareness are obligatory for the work with patients, persons close to them or bereaved persons. Regarding recruitment strategies and interview situations, close attention has to be paid to ethical considerations and anticipation of problematic situations. This was accounted for in the selection of research staff members and in preparing staff and written materials.

The strength of the study is that it integrates different perspectives. Due to that fact, implementation, acceptance, and usefulness of the outcome-set in routine health care might be facilitated for patients and providers. This should facilitate the implementation, acceptance, and usefulness of the outcome-set in routine health care for both – patients and providers. A further strength is the integration of different methods and a multi-step process within the developing and testing protocol as suggested by Campbell et al. [32]. This includes an overview of existing literature, participatory observations, qualitative interviews, focus groups, routine data analysis, a pilot-study, and a statewide implementation.

However, some limitations have to be mentioned. The study in the state of Hesse takes place under very good conditions: As mentioned above, all SOPC team are organized within the Professional Association of SOPC of Hesse. Therefore, external validity might be limited. Finally, all of the instruments are meant for adult patients only. The needs of young adults and children might differ from the needs from adult patients and therefore have to be examined separately [9].

Provided that our study shows feasibility, utility, and acceptance in participants, and that the instruments show validity and reliability, the consequence of this study could be a nationwide implementation (in Germany).

The results of this study could improve the quality of the care process and help to compare patient groups, care services and regions in a valid way. The reporting of patient-relevant parameters could also contribute to an optimization and advancement of the SOPC directive in Germany. The standardized and validated measurement approach consists of internationally recommended and prevalent instruments. Therefore, this study might contribute to the necessity of stipulating documentation and to establish a profound scientific basis to research standards for primary outcomes in outpatient palliative care internationally. This is very important because there is a great diversity in outcome measures used in SOPC [8, 33].

## Abbreviations

CQ-index PC: Consumer quality index palliative care
EAPC: European Association for Palliative Care
ECOG: Eastern Cooperative Oncology Group Performance Status
ELSAH: Evaluation of Specialized Outpatient Palliative Care in the German state of Hesse
GAS: Goal attainment scaling
G-BA: Gemeinsamer Bundesausschuss (Federal Joint Committee)
IPOS: Integrated palliative care outcome scale
OACC: Outcome assessment and complexity collaborative
PC: Palliative care
PROM: Patient reported outcome measure
SEC: Sense of security
SOPC: Specialized outpatient palliative care
VOC: IPOS Views on Care
ZBI: Zarit burden interview
